# Vascular Access Site Complications Do Not Correlate With Large Sheath Diameter in TAVI Procedures With New Generation Devices

**DOI:** 10.3389/fcvm.2021.738854

**Published:** 2021-12-08

**Authors:** Birgid Gonska, Christopher Reuter, Johannes Mörike, Wolfgang Rottbauer, Dominik Buckert

**Affiliations:** Department of Internal Medicine II–Cardiology, University of Ulm, Ulm, Germany

**Keywords:** transcatheter aortic valve implantation, vascular complications, bleeding, sheath size, calcification

## Abstract

**Background:** Vascular complications after transfemoral transcatheter aortic valve implantation (TAVI) are associated with morbidity and mortality. However, consistent predictors have not been identified yet. The size of the implantation sheath seems to play a role, though especially with new generation TAVI devices and their improved sheaths and delivery systems this remains uncertain.

**Objectives:** This study aimed to determine the incidence and predictors of access site-related vascular complications (VC) in the era of new generation TAVI devices.

**Methods and Results:** Four hundred consecutive patients receiving TAVI in an experienced tertiary care center were analyzed. VC occurred in 89 patients (22.25%) with the majority being minor VC (21%) and only 1.25% major VC. Possible predictors for VC were tested, and only peri-interventional dual antiplatelet therapy (DAPT) showed to be predictive for VC [OR 2.11 (95% CI 1.10–4.06, *p* = 0.025)]. The female gender [OR 0.75 (95% CI 0.44–1.3), *p* = 0.31], sheath to femoral artery ratio >1.05 [OR 1.18 (95% CI 0.66–2.08, *p* = 0.58)], calcification of the access site vessel [OR 0.83 (95% CI 0.48–1.42, *p* = 0.48)], known peripheral artery disease [OR 0.95 (95% CI 0.4–2.25, *p* = 0.9)], and BMI ≥ 25 kg/m^2^ [OR 0.69 (95% CI 0.41–1.19, *p* = 0–19)] were not predictive of VC. The larger sheath with 20 French even showed less VC than the smaller sheath with 16 French [OR 0.43 (95% CI 0.25–0.74, *p* = 0.002)].

**Conclusions:** Overall, the rate of major and minor VC was low in this study population (for major VC: rate of 1.25%). Predefined risk factors were not associated with the occurrence of VC, except for peri-interventional treatment with DAPT. Especially, larger sheath size could not be identified as a predictor for VC in the setting of TAVI procedures performed with contemporary devices.

## Introduction

Since the first transcatheter aortic valve implantation (TAVI) in 2002 by Cribier et al. (1), it has become the standard of care for inoperable patients or patients at high risk for surgical valve implantation (2). It has grown into a rapidly evolving alternative for patients at intermediate risk and is even progressing toward low-risk patients (3, 4).

The preferred approach for TAVI is transfemoral access, which has been associated with a better outcome than non-transfemoral access (5). With the first-generation TAVI devices, major vascular complications (VC) occurred in ~10% of patients (6–10). The occurrence of major VC proved to be associated with higher rates of morbidity and mortality (6, 11–13).

The growing experience with the management of percutaneous vascular access, development of new percutaneous suture devices, and improvements of the TAVI devices including implantation sheaths and delivery catheters have already led to a decline of VC compared with the beginning of TAVI. Nonetheless, VC is still one of the more common complications after transfemoral TAVI with incidences of 2 to 10% for major VC, and a wide range of 2 to 29% for minor VC (12, 14–16).

In previous studies, a variety of risk factors have been identified to influence the occurrence of VC after transfemoral TAVI, such as female gender, obesity, peripheral artery disease (PAD), femoral artery diameter, sheath size or sheath to (ilio-)femoral artery ratio (SIFAR; SFAR, respectively), and calcification of the access site vessel and center experience (7, 13, 17–20).

This study aimed to analyze the incidence of access site-related VC in an all-comers patient cohort treated with the newest generation TAVI devices and to evaluate whether risk factors can be identified that have an impact on the occurrence of VC. Therefore, we assessed access site-related VC as defined by the updated standardized endpoint definitions for TAVI according to the second Valve Academic Research Consortium- (VARC-2) criteria in patients treated with the newest generation TAVI devices (21). We chose to analyze TAVI devices with non-expandable sheaths to ensure consistent sheath diameters: the Boston Scientific Lotus Edge (BLE) valve (20 French) and the Medtronic Evolut Pro/R (MEV) valve with a low-profile delivery system (EnveoPro, 16 French).

## Methods

In this retrospective single-center study, 611 consecutive patients treated with transfemoral TAVI for aortic valve disease between January 2019 and May 2020 were screened for treatment with either the self-expandable MEV Pro (size 23/26 or 29 mm) and MEV R (size 34 mm) or with the mechanically expandable BLE.

The decision for transfemoral TAVI was made by the interdisciplinary heart team according to the 2017 European Society of Cardiology/ European Association for Cardio-Thoracic Surgery Guidelines for the management of valvular heart disease (22). The study was approved by the local ethics committee. All patients gave written informed consent.

**Figure 1:**
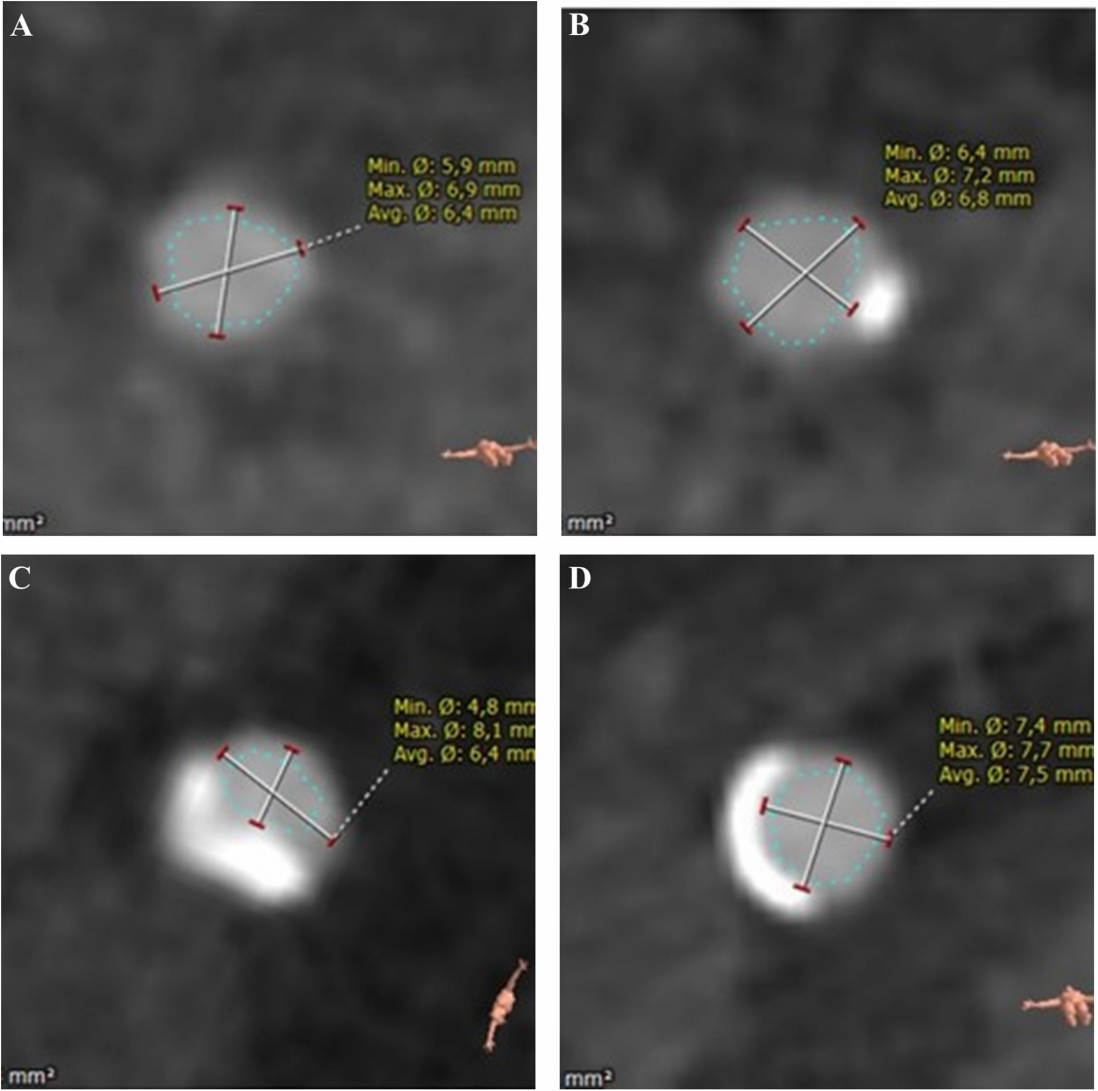
Quantification of calcification of common femoral artery (derived from CT): none **(A)** mild **(B)** severe **(C,D)**.

All patients underwent preprocedural 256 multislice contrast-enhanced CT, which was evaluated with a dedicated software (3mensio Structural Heart 9.1 software, Pie Medical Imaging B.V., Maastricht, The Netherlands). Besides the decision for the valve size, there was also an evaluation of the vascular access. The slice thickness for the evaluation of vascular access was 0.7 to 1.0 mm. Routinely the access site was determined preprocedural and the size of the common femoral artery (CFA) was measured at the expected puncture position. Furthermore, calcification was semi-quantitatively classified in none, mild (calcification of not more than 25% of the circumference and not relevant protrusion into the lumen), or severe (calcification of more than 25% of the circumference, more than two spots, or relevant protrusion into the lumen) (Figure 1).

According to the hospital protocol, antiplatelet therapy was continued peri-interventionally whereas oral anticoagulation was stopped pre-procedurally. In these patients, single antiplatelet therapy was used peri-interventionally.

The decision for BLE or MEV device was made according to the experienced interventional cardiologist. Formally the BLE introducer set requires access vessel diameters of 6.5 mm or larger, the EnveoPro delivery system 5.5 mm or larger.

Transcatheter aortic valve implantation (TAVI) was performed in a hybrid catheterization laboratory under conscious sedation by an experienced operator team of four interventionalists with a standardized procedure protocol. First, the puncture of the non-access site CFA was performed under fluoroscopic control, and then the access site was cannulated under angiographic visualization *via* cross-over angiography from the non-access site, attempting for the puncture height predefined by the CT measurements. There was no use of ultrasound for the puncture. After the insertion of a 6 French sheath, angiography in an ipsilateral oblique view was performed to control the exact position of the puncture. Afterward, the vascular closure device was applied. For that matter, two Perclose ProGlide devices (Abbott Vascular, Santa Clara, California) were used at a 2-h angle (eleven o'clock and one o'clock). Then, the TAVI sheath was inserted over a stiff wire and heparin was administered to achieve an activated clotting time of 250–300 s. For the MEV Pro 23-, 26-, and 29-mm valve as well as for the MEV R 34 mm valve a 16 French Sheath (Cook Check-Flo Performer Introducer; Cook Medical, Limerick, Ireland) was used and later exchanged for the 16 French equivalent EnveoPro delivery system with an inline sheath, loaded with the valve. For the BLE the 20 French Boston Lotus Introducer sheath was inserted which remained in place throughout the procedure.

Before removal of the access site sheath, crossover access was obtained from the non-access femoral site with a 6 French pigtail catheter placed in the external iliac artery of the access site. Then, the access site sheath was removed and the ProGlides knots were pushed down and locked. Afterward, an angiography of the access site was performed. If necessary, dependent on the result of the angiogram and the discretion of the operator, either endovascular therapy (covered stent, percutaneous transluminal angioplasty) or manual compression was used to control the access site in case of a leak. Closure of the non-access site was achieved by 6 French Angio-Seal devices (Terumo Europe N.V., Leuven, Belgium), or in case of use of a covered stent at the access site requiring a sheath size of 8–10 French by one ProGlide. Postprocedural, there was a clinical evaluation of the access and non-access site. Any hematoma of the access site was documented. Patients with periprocedural findings other than none or mild leak or patients with suspicious findings in the postprocedural clinical examination had Doppler-/Duplex ultrasound evaluation of access site and non-access site. If this showed pseudoaneurysm, either manual compression therapy was used or an injection of thrombin.

**Figure 2:**
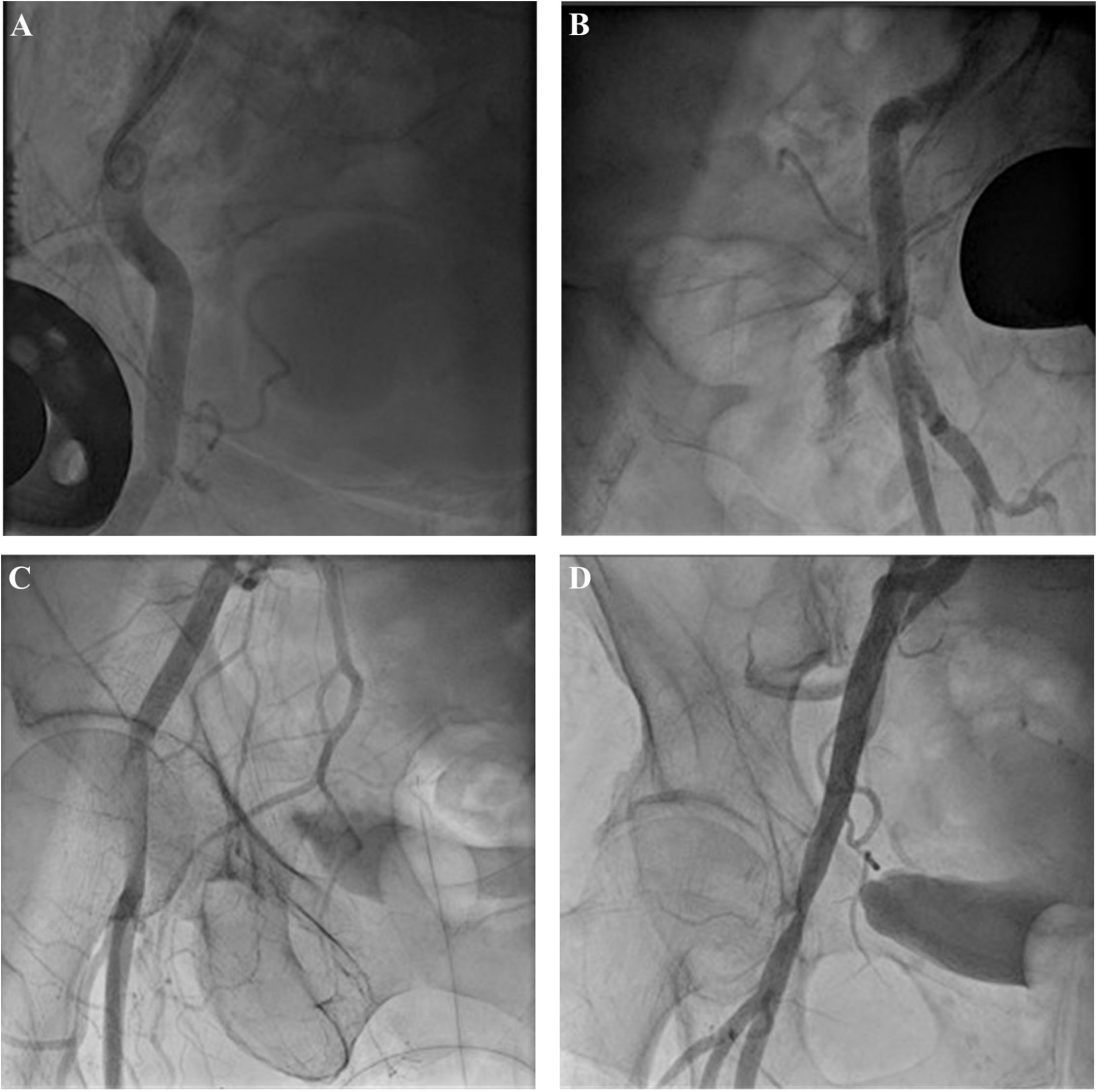
Evaluation of angiogram after use of closure device: minor leak **(A)**, severe leak **(B)**, dissection **(C)**, and stenosis with minor leaks **(D)**.

The last angiogram of the access site was evaluated retrospectively concerning any sign of leaks, dissection/endovascular flap, stenosis (lumen reduction of ≥50%), and total occlusion of the CFA (Figure 2).

The baseline characteristics of the patients, including medication and relevant medical history, were documented, as well as the clinically relevant periprocedural data. We assessed the sheath to femoral artery ratio (SFAR) by dividing the outer diameter of the sheath (6.667 mm for the Medtronic system and 7.9 mm for the Boston Lotus system) by the diameter of the CFA at the expected puncture position of the access site, evaluating SFAR using the minimal diameter as well as the mean diameter.

The primary endpoint of this analysis was the predefined VARC-2 endpoints vascular access site and access-related complications with the focus on the access site itself. Major VCs at the access site were defined as vascular injuries such as perforation, bleeding, hematoma, dissection, stenosis, or pseudoaneurysm, leading to death, life-threatening or major bleeding. Minor VCs were assessed at the access and non-access site and defined as vascular injury as mentioned for the major VC however not leading to death, life-threatening, or major bleeding. Due to the small number of major VCs, patients with minor or major VC were combined and compared with patients without VC.

Furthermore, clinically relevant VARC-2 defined endpoints pacemaker, bleeding, peri-interventional stroke/transitory ischemic attack (TIA), and device success were assessed.

### Statistical Analysis

We performed a sample size calculation based on assumed incidences for the small and the large sheath. Chen et al. had evaluated predictors for suture device failure and vascular complications in a patient cohort of 458 patients with interventions with sheath sizes 16 to 26 French (18). The study showed a significantly higher incidence of suture device failure and following vascular complications with the use of >21 French sheath size than the ≤ 21 F sheath size with 17.2% vs. 4.9%, *p* < 0.001; using a receiver operating characteristic (ROC) curve sheath, size ≥19 French was found to be significantly associated with suture device failure. Barbanti et al. had experienced comparable results in their study on 375 patients (≥19 French rates of major VC 17.5 vs. 5.9%, *p* < 0.001) (20). Based on these results with expected event rates of 15% for the 20 French and 5% for the 16 French access and a power of 80% and an α-level of 0.05 group sizes of 141 patients each were found to be adequate. However, to achieve a sufficient safety margin we aimed for 200 consecutive patients with each valve type/sheath size. Statistical analysis was performed with the MedCalc software (MedCalc Version 19.6, MedCalc Software Ltd, Ostend; Belgium). Continuous variables are expressed as mean ± one SD and were compared with the *t*-test. Categorical variables are presented as counts and percentages and differences between proportions were calculated by using the χ^2^ test. A logistic univariate and multivariate regression analysis were performed to identify predictors for VC after TAVI, results presented as odds ratio with 95% confidence interval (CI). A value of *p* < 0.05 was considered statistically significant.

## Results

**Figure 3:**
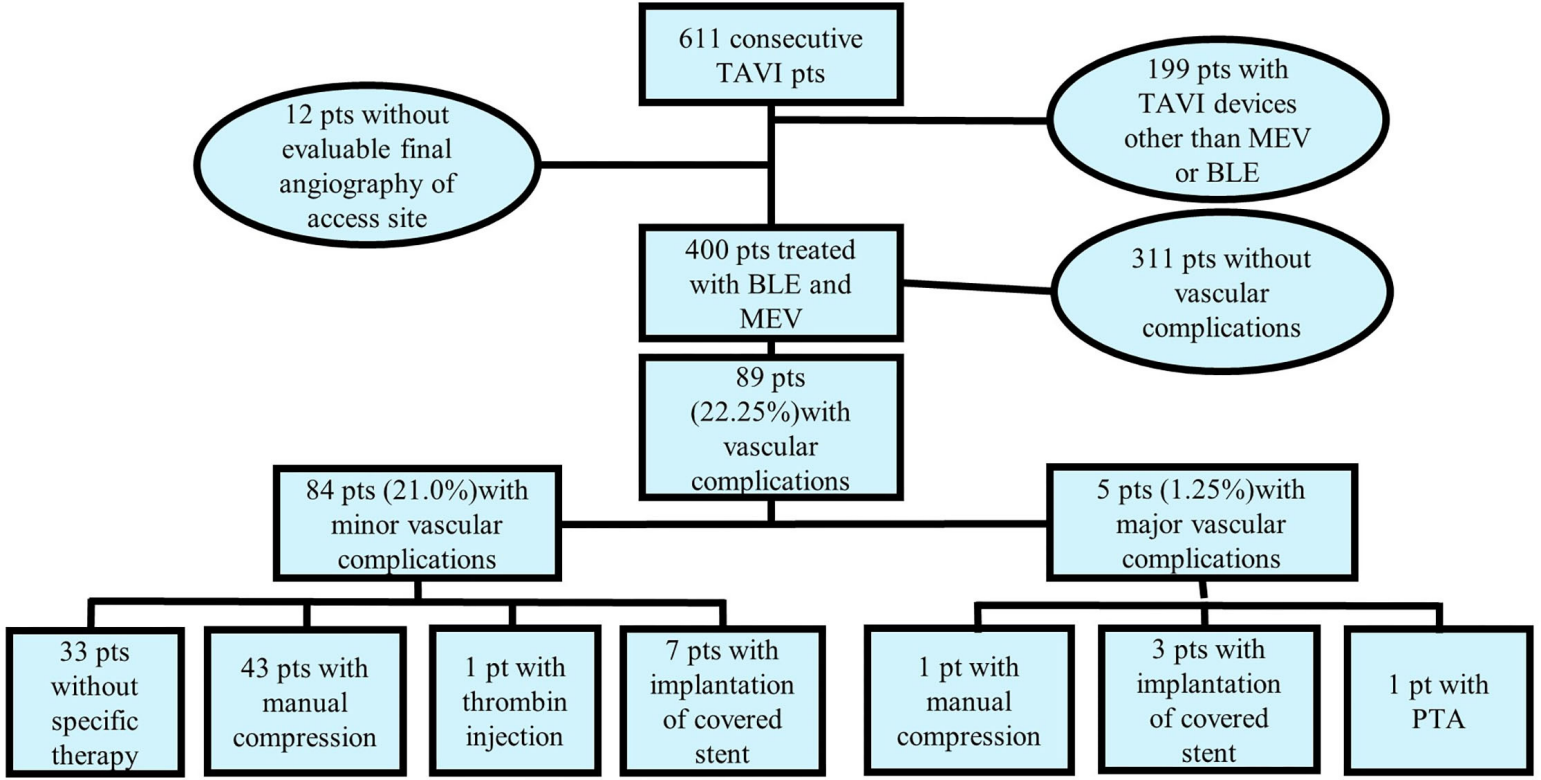
Treatments for patients with minor and major access site vascular complications.

In all 400 patients, an aortic valve prosthesis was successfully implanted, in 399 patients one valve, in one patient there was the implantation of a second valve due to an embolized MEV, without further complications. There was no conversion to surgery and there was no periprocedural death. The rate of major VC was low with only 1.25% (5 patients). Two of these patients showed severe leak and three showed severe leak and dissection of the CFA after use of the vascular closure devices. Three patients were treated with the implantation of a covered stent, one patient had percutaneous transluminal angioplasty (PTA) only of the CFA and one patient was treated with manual compression only (Figure 3). All of these patients met the criteria of major bleeding complications according to the VARC-2-criteria, even though only three of them showed hematoma of the access site afterward. Patients with major VC had a significantly larger drop in hemoglobin after the TAVI procedure (3.78 ± 1.93 vs. 1.34 ± 1.15 g/dl, *p* = 0.048), and received significantly more units of blood (0.75 ± 0.96 units of blood vs. 0.05 ± 0.34, *p* < 0.001). Minor vascular complications were more frequent with 21% (84 patients). Most of these patients had access site hematoma (66 patients), four patients developed pseudoaneurysm, and in 12 patients there was stenosis of at least 50% of the CFA by duplex ultrasound without need for further treatment. There were no major VC at the non-access site, only minor VC (69 patients, 17.25%), the majority having a hematoma (57 patients), nine developing pseudoaneurysms, which was treated with thrombin injection or manual compression (6 patients and 3 patients, respectively), and 3 patients with stenosis of at least 50% of the CFA by duplex ultrasound without need for further treatment.

### Baseline Characteristics

The baseline characteristics of the patients with and without VC are displayed in Table 1. There was no statistically significant difference concerning their medical history or clinical presentation. There was especially no difference in gender (female 51.1% in no-VC vs. 56.2%, *p* = 0.1), in body mass index (27.4 ± 5.1 kg/m^2^ in no-VC group vs. 27.5 ± 4.5 kg/m^2^ in VC group, *p* = 0.86) or known history of PAD (9% in both groups). There was a trend of more patients being treated with peri-interventional dual antiplatelet therapy in the cohort of patients with VC (21.3 vs. 13.8%, *p* = 0.08).

**Table 1:** Baseline clinical characteristics.

	**No VC *n* = 311**	**VC *n* = 89**	***P*** **-Value**
Age, years	80.5 ± 6.3	79.6 ± 6.3	0.24
Female	159 (51.1%)	50 (56.2%)	0.40
BMI (kg/m^2^)	27.4 ± 5.1	27.5 ± 4.5	0.86
Diabetes mellitus	79 (25.4%)	28 (31.5%)	0.26
Chronic renal failure with dialysis	5 (1.6%)	2 (2.2%)	0.70
Coronary artery disease	164 (52.9%)	54 (60.7%)	0.20
History of myocardial infarction	28 (9.0%)	10 (11.2%)	0.71
History of cardiac surgery	24 (7.7%)	10 (11.2%)	0.30
Known PAD	28 (9.0%)	8 (9.0%)	1.00
History of stroke or intracerebral bleeding	46 (14.8%)	11 (12.4%)	0.56
Pulmonary disease	126 (40.5%)	43 (48.3%)	0.20
NYHA class III/IV	207 (66.6%)	65 (73%)	0.25
Ejection fraction (%)	51.1 ± 11.0	52.6 ± 9.0	0.26
STS PROM	3.48 ± 2.37	3.52 ± 2.58	0.89
**Medication at baseline**			
ASA only	135 (43.4%)	42 (47.2%)	0.53
DAPT	43 (13.8%)	19 (21.3%)	0.08
Oral anticoagulation	109 (35.5%)	22 (24.7%)	0.06

*Values are mean ± SD or n (%).*

*BMI, Body mass index; PAD, peripheral artery disease; NYHA, New York Heart Association; STS-PROM, Society of Thoracic Surgeons predicted risk of mortality; ASA, acetylsalicylic acid; DAPT, dual antiplatelet therapy*.

### CT Evaluation of Access Site Vessel

The evaluation of the preprocedural CT data revealed no significant difference in the diameter of the access vessel in patients treated with the BLE (7.5 ± 1.23 mm) or the MEV (7.36 ± 1.51 mm, *p* = 0.30). Seventeen patients (8.5%) treated with MEV had a vessel diameter smaller than the formally recommended 5.5 mm, 36 (18%) treated with BLE a had vessel diameter smaller than the recommended 6.5 mm. Patients with and without VC exhibited no relevant differences between the groups concerning access vessel characteristics as well (Table 2). Thirty-six percent of the patients without VC had calcification of the access vessel and 31.5% for the patients with VC (*p* = 0.43).

**Table 2:** Characteristics of access site common femoral artery.

	**No VC *n* = 311**	**VC *n* = 89**	***P*** **-Value**
Minimal diameter of CFA, mm	6.73 ± 1.21	6.58 ± 1.69	0.36
Maximum diameter or CFA, mm	8.17 ± 1.47	8.08 ± 1.74	0.65
Mean diameter of CFA, mm	7.45 ± 1.29	7.33 ± 1.68	0.49
SFAR (mean diameter)	1.01 ± 0.20	1.02 ± 0.29	0.74
SFAR (minimal diameter)	1.13 ± 0.24	1.18 ± 0.55	0.18
SFAR≥1.05 (mean diameter)	120 (38.7%)	33 (37.5%)	0.84
SFAR≥1.05 (minimal diameter)	200 (64.5%)	50 (56.8%)	0.19
**Calcification of CFA**			
None	199 (64.0%)	61 (68.5%)	0.64
Moderate	78 (25.1%)	18 (20.2%)	
Severe	34 (10.9%)	10 (11.2%)	

*Values are mean ± SD or n (%).*

*CFA, common femoral artery; SFAR, sheath to femoral artery ratio*.

### Procedural Data

**Figure 4:**
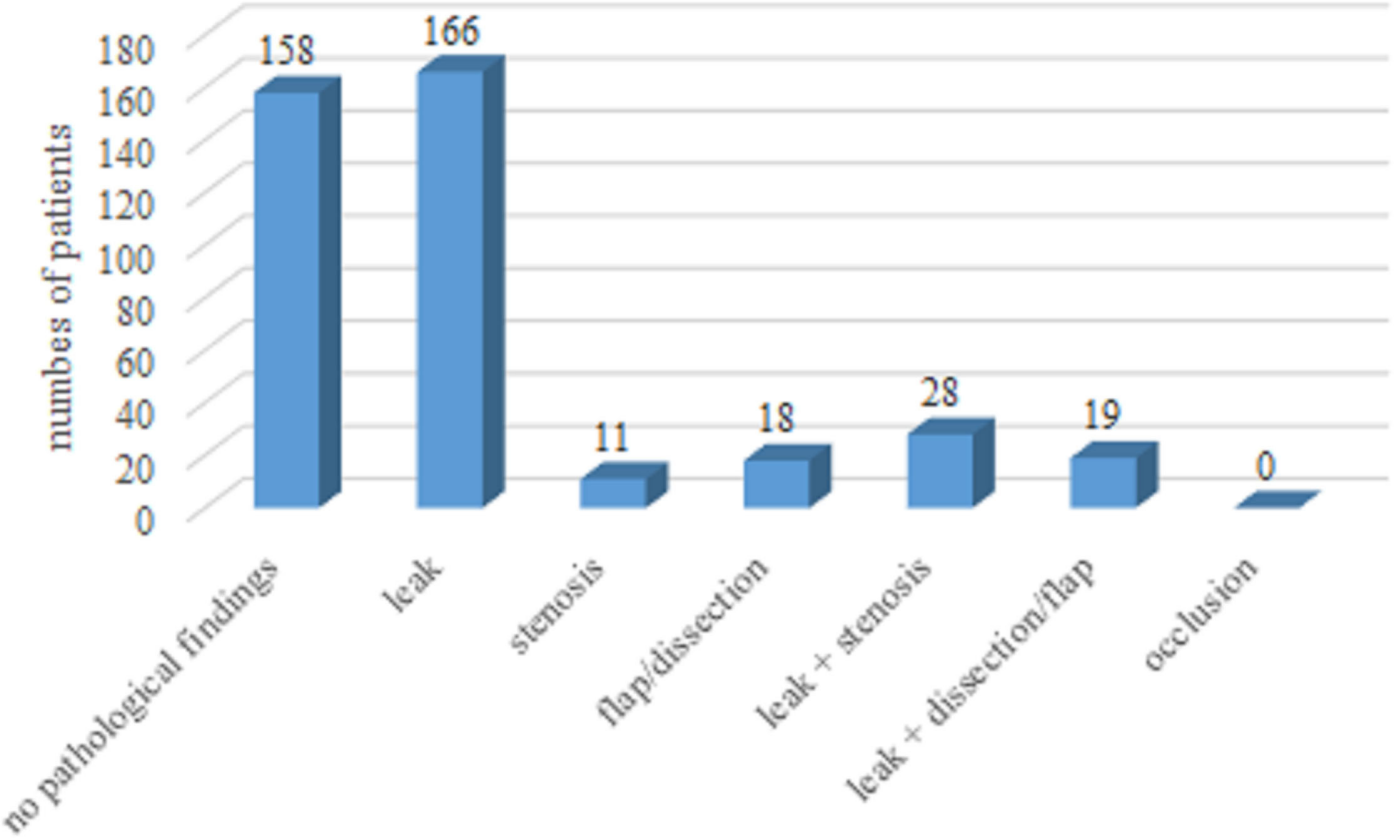
Results of the angiogram of the access site after use of the closure device.

The most frequently implanted size of BLE was 25 mm (43%), followed by 27 mm (38%), and 23 mm (19% of BLE). For the MEV, the most frequently implanted size was 29 mm (35.5%) followed by 26 mm (29%), 34 mm (28%), and 23 mm (7.5%). The procedural data with respect to VC are shown in Table 3. Patients receiving MEV had minor VC more often (64 vs. 36% *p* = 0.003), whereas major VC differed only numerically without statistical significance (2 vs.0.5%, *p* = 0.18). In relation to the difference in valve type distribution concerning VC outer sheath/delivery, the catheter diameter was smaller in patients with VC. SFAR however was not associated with VC in the study cohort (*p* = 0.74 for SFAR derived from mean CFA diameter; *p* = 0.18 for SFAR derived from minimal CFA diameter.) The results of the final angiography of the access site after use of the closure device (and before possible intervention are displayed in Figure 4. A leak of any sort was quite frequent with the 41.5%, however, the results of the angiography were not predictive for VC.

**Table 3:** Procedural data.

	**No VC *n* = 311**	**VC *n* = 89**	***P*** **-Value**
**Valve type**			
MVE	143 (46.0%)	57 (64.0%)	0.003
BLE	168 (54.0%)	32 (36.0%)	
Outer diameter of sheath/delivery catheter, mm	7.33 ± 0.62	7.11 ± 0.60	0.003
**Angiography of access site**			
Leak	159 (51.1%)	53 (59.6%)	0.16
Dissection/flap	45 (15.4%)	15 (17.2%)	0.26
Stenosis/occlusion	7 (2.3%)	4 (4.5%)	0.26

*Values are mean ± SD or n (%).*

*MVE, Medtronic evolut; BLE, Boston lotus edge; SFAR, Sheath to common femoral artery ratio*.

### Postprocedural Data

Bleeding complications occurred in 50 patients (12.5%), most of them being minor bleedings (33 patients, 8.25%) due to access site or non-access site hematoma. Twelve patients developed major bleedings (3%) and five patients had life-threatening bleedings, none of these access sites or non-access sites related there being two periprocedural intracerebral bleedings and three hemorrhagic pericardial effusions after pacemaker implantation after TAVI. Due to the definition of vascular complications minor and major bleeding complications occurred significantly more often in the group of patients with VC. The postprocedural pacemaker implantation rate was 19.25% for the overall population, significantly more frequent after treatment with the BLE (25%) compared with the MEV (13.5%, *p* = 0.004). The rate of device success was high at 98%. Data on postprocedural outcomes concerning VC are presented in Table 4.

**Table 4:** Procedural outcome.

	**No VC *n* = 311**	**VC *n* = 89**	***P*** **-Value**
Device success	305 (98.1%)	87 (97.8%)	0.85
Periprocedural stroke/TIA	10 (3.3)	4 (4.5%)	0.56
Pacemaker implantation	67 (21.5%)	10 (11.2%)	0.03
**Bleeding complications**			
Minor bleeding	17 (5.5%)	16 (18.0%)	0.0002
Major bleeding	6 (1.9%)	6 (6.7%)	0.02
Life threatening bleeding	4 (1.2%)	1(1.1%)	0.90

*Values are mean ± SD or n (%).*

*TIA, transitory ischemic attack*.

### Logistic Regression Analysis

The parameters that were distributed significantly different between groups or had been identified to be predictive of VC in previous studies such as age, gender, body mass index (BMI), PAD, large sheath size, SFAR, calcification, and DAPT were entered into a univariate and multivariate logistic regression analysis (Table 5). Peri-interventional treatment with DAPT showed to be an independent predictor for VC (OR 2.11, 95% CI 1.10–4.06, *p* = 0.025). Large sheath size was not independently associated with a higher rate of vascular complications.

**Table 5:** Predictors of vascular complications.

	**Univariate analysis**		**Multivariate analysis**	
	**OR (95% CI)**	***P*** **-Value**	**OR (95% CI)**	***P*** **-Value**
Age	0.98 (0.94–1.01)	0.24	0.98 (0.94–1.02)	0.30
Gender (female)	0.82 (0.51–1.31)	0.40	0.75 (0.44–1.30)	0.31
BMI	1.00 (0.96–1.05)	0.86	–	–
BMI ≥25 kg/m^2^	0.69 (0.41–1.15)	0.14	0.69 (0.41–1.19)	0.19
SFAR (mean diameter)	1.19 (0.42–4.0)	0.74	–	–
SFAR > 1.05 (mean diameter)	0.95 (0.58–1.55)	0.84	1.18 (0.66–2.08)	0.58
Calcification of CFA	0.82 (0.49–1.35)	0.42	0.83 (0.48–1.42)	0.48
Known PAD	1.0 (0.44–2.28)	1.0	0.95 (0.40–2.25)	0.90
DAPT	1.69 (0.93–3.08)	0.09	2.11 (1.10–4.06)	0.025
20French sheath	0.48 (0.30–0.78)	0.0025	0.43 (0.25–0.74)	0.002

*OR, odds ratio; CI, 95% confidence interval; BMI, body mass index; SFAR, sheath to femoral artery ratio; CFA, common femoral artery; PAD, peripheral artery disease; DAPT, dual antiplatelet therapy*.

## Discussion

With the use of the newest generation TAVI devices, MEV and BLE, we report a low rate of access site-related major VC with only 1.25%. Facing the low event rate, specific predictors of major VC could not be determined in this study. For the whole entity of VC combining major and minor VC peri-interventional treatment with DAPT showed to be an independent predictor for the occurrence of VC. We could not verify any of the other previous studies that identified predictors of VC.

The data on VC with the newer generation TAVI devices are still limited, most of the studies have evaluated the balloon-expandable valve with its expandable sheath. There are only a few studies on the MEV Pro valve: Major VC ranging from 0 to 10% in the first two studies, with only 60 and 74 patients, however, the largest published study cohort with 629 patients treated with the MEV Pro (and thus the EnveoPro delivery system) had a rate of only 3.3% major VC (15, 16, 23). Concerning the BLE valve there are no comparable data published yet, however, rates of major VC with its predecessor, the Boston Lotus valve were slightly higher ranging from 2.9 to 7.5% (23, 24). Our even lower rate of major VC in this study could be explained by the fact that the evaluated study cohort recently treated between 2019 and 2020, consisted of a lower risk cohort with a Society of Thoracic Surgeons–predicted risk of mortality (STS-PROM)-score <4%, furthermore, the study center being a high volume TAVI center with more than 400 procedures per year with a consistent team of experienced operators. In an analysis from the France TAVI Registry, Beurtheret et al. found a decline in major vascular complications between the period 2013–2015 and 2016/2017 of 1.44 to 1.02% (*p* = 0.005) and referring this to the increase in performed TAVI procedures from the first to the second period (25).

Despite the low rate of major VC, access site-related minor VC was still frequent with 21%., because of a high rate of minor VC, mainly due to local hematoma occurring in the postprocedural period. The incidence of minor VC varies widely in the literature from 2 to almost 30% if mentioned at all in publications. For studies with retrospective analysis, this could be explained by center-specific differences in thoroughness on documentation of clinically non-significant access site hematoma, which already accounts as minor VC according to the VARC-2 criteria. In contrast to the negative impact of major VC on mortality, there is no evidence, that minor VC is associated with increased mortality or increased length of hospital stay (8).

### Vessel Size/Sheath Size

In TAVI studies or studies about large endovascular access for percutaneous procedures, a variety of parameters have been indicated to influence the occurrence of VC. The most obvious seems to be the size of the access vessel, respectively, sheath size, and sheath to access site vessel ratio. Especially the ratio of the sheath to the (ilio)-femoral vessel at the access site has been described as a predictor for major VC. In the study by Hayashida et al., an SFAR threshold of 1.05 was found to be predictive for major VC, in the study by Toggweiler et al., a threshold of 1.0 (7, 8). For the newer generation, TAVI devices S(I)FAR has been confirmed as a predictor for major VC by van Kesteren et al. for the balloon-expandable Edwards Sapien 3 valve and its expandable sheath [unadjusted OR 7.51 (1.61–34.95), *p* = 0.01)], though the area under the curve was much lower in comparison to studies describing S(I)FAR with older generation TAVI devices, indicating poorer accuracy (13). Potluri et al. also experienced SFAR as independently associated with VC, again, a study with the Edwards Sapien 3 as the most commonly used device with its expandable sheath (19). To our knowledge, there are no published data on predictors of VC concerning the ratio of the sheath to the access site vessel regarding new generation non-expandable sheaths. In contrast to previous studies, especially the large meta-analysis by Ueshima et al. we evaluated a recently treated low-risk patient cohort with low STS-PROM score, low rate of PAD, and use of new generation non-expandable sheaths, which might have led to the fact, that we did not experience higher rates of VC in patients with the use of the larger sheath or a higher SFAR (17). Furthermore, we only used the ProGlide vascular closure device, whereas, in older studies, other vascular closure devices as the Prostar (Abbott Vascular, Santa Clara, CA, USA) had still been in use. Mehilli et al. (12) and Seeger et al. (10) could both show in their studies that there were significantly fewer VC complications and bleeding complications with the use of ProGlide in comparison to the Prostar device.

A certain bias of not treating patients with more complex vascular access routes and smaller diameters with the larger sheath TAVI device cannot be completely excluded since the choice of the used TAVI device was not defined by a prospective randomized study design. However, there was no significant difference in vessel diameters concerning the CFA between the two valve types. Interestingly, the logistic regression analysis showed the larger sheath size to be associated with less VC, however, that statement has its limitations since this was contrary to the initial approach on this study and its power calculation. A further explanation could lay within the necessity of removal of the sheath for the smaller sheath size (MEV) before insertion of the delivery system with subsequent intermittent manual compression.

Throughout the published studies on VC, the measurement of SFAR does not seem to be consistently defined. In some studies, the reference for the sheath diameter is the inner diameter, in others the outer diameter. This is correlated to the mean or the minimal diameter of the access vessel or even concerning the smallest diameter of the complete iliofemoral vessel length.

### Calcification

There is no unified definition/quantification of access vessel calcification yet. We tried to construct a simple semi-quantitative definition based on the CT data. However, we failed to identify severe calcification or calcification in general of the CFA as a predictor for major or minor VC. Some older reports have demonstrated that iliofemoral calcification, assessed semi-quantitatively and slightly different in every report, is predictive of major VC (7). However, newer reports with more detailed CT evaluation of calcification, as Fonseca et al. who defined specific calcium thresholds, could not confirm this (14).

### Periprocedural Medication

Dual antiplatelet therapy (DAPT) is known to increase bleeding risk in general and in TAVI, if that translates to a higher risk of VC is still not evident. Hioki et al. evaluated 540 TAVI patients and found DAPT to be a significant predictor for bleedings, however, this did not translate into a higher rate of VC, with only approximately one-third of bleedings being associated with the access site (26). In our study cohort, peri-interventional treatment with DAPT was independently associated with a higher rate of VC.

### Peripheral Artery Disease

The presence of PAD seems to be an obvious parameter for the occurrence of VC, though existing data are not consistent. In previous studies, that have identified PAD as a predictor for VC, the rate of VC usually varied around 20% (13, 17). In our study, we could not confirm the influence of that parameter, however, we only saw a low rate of reported PAD with 9% in our study cohort, as well as Fonseca et al. who described a rate of 11.4% and could not identify PAD as a predictor for VC as well (14). The low reported incidence of reported PAD in our cohort may be attributed to the cohort being a low-risk cohort with a mean STS-PROM of <4%.

## Limitations

Our study has several limitations. First, it is a retrospective single-center analysis. The lack of randomization may influence the selection of patients and outcomes. As for the documentation of minor vascular complications, not every patient had a duplex ultrasound of the access site post procedurally, only those with already clinically suspected pathological findings. Therefore, it is possible to have overlooked some minor VC complications.

The study was powered for the parameter sheath size and a difference of at least 10%. Therefore, we cannot derive definitive conclusions on other parameters, and cannot exclude, that smaller differences in predicting values of sheath diameter are present and are not detected for power limitations.

## Conclusion

With the newest generation of TAVI devices, major VC seems to have reached the bottom line. The incidence of VC was low and most of the previously detected potential risk factors showed no relevant influence on the occurrence of VC in general in our study population of 400 patients, neither on major VC and thus did not offer potential angles for optimization. Only peri-interventional treatment with DAPT was associated with the occurrence of VC. Most likely, these low major VC rates are nowadays achieved by device improvements concerning sheaths and delivery systems, profound knowledge of performing percutaneous vascular access with large diameters, and well-established endovascular treatment options in case of need.

## Data Availability Statement

The raw data supporting the conclusions of this article will be made available by the authors, without undue reservation.

## Ethics Statement

The studies involving human participants were reviewed and approved by Ethics Committee of the University of Ulm, Ulm Germany. The patients/participants provided their written informed consent to participate in this study.

## Author Contributions

BG: conceptualization, investigation, validation, methodology, formal analysis, and writing-original draft. CR and JM: investigation and data curation. WR: funding acquisition conceptualization, investigation, supervision, writing-review, and editing. DB: conceptualization, investigation, methodology, formal analysis, supervision, writing-review, and editing. All persons who meet the authorship criteria are listed as authors, certify that they have participated sufficiently in the work to take public responsibility for the content, including participation in the concept, design, analysis, writing, or revision of the manuscript, and provided critical feedback to the manuscript and approved the final version of the manuscript.

## Funding

For the conduction of the study, the Department of Internal Medicine II, University of Ulm, received restricted funds from Medtronic (Medtronic Minneapolis, Minnesota, USA). The funder was not involved in the study design, collection, analysis, interpretation of data, the writing of this article, or the decision to submit it for publication.

## Conflict of Interest

The authors declare that the research was conducted in the absence of any commercial or financial relationships that could be construed as a potential conflict of interest.

## Publisher's Note

All claims expressed in this article are solely those of the authors and do not necessarily represent those of their affiliated organizations, or those of the publisher, the editors and the reviewers. Any product that may be evaluated in this article, or claim that may be made by its manufacturer, is not guaranteed or endorsed by the publisher.
